# What are the risk factors for postoperative infections of third molar extraction surgery: A retrospective clinical study-?

**DOI:** 10.4317/medoral.22556

**Published:** 2018-12-24

**Authors:** Shintaro Sukegawa, Kyoko Yokota, Takahiro Kanno, Yoshiki Manabe, Yuka Sukegawa-Takahashi, Masanori Masui, Yoshihiko Furuki

**Affiliations:** 1 DDS, PhD. Consultant, Division of Oral and Maxillofacial Surgery, Kagawa Prefectural Central Hospital, 1-2-1, Asahi-cho, Takamatsu, Kagawa 760-8557, Japan; 2 MD, MSc. Director, Division of Infectious Disease, Kagawa Prefectural Central Hospital Kagawa, Japan; 3 DDS, PhD, FIBCSOMS, FIBCSOMS-ONC/RECON. Associate Professor & Director, Department of Oral and Maxillofacial Surgery, Shimane University Faculty of Medicine, Shimane, Japan; 4 PhD. Admission Center, Kagawa University, Takamatsu, Kagawa, Japan; 5 DDS. Clinical Fellow, Division of Oral and Maxillofacial Surgery, Kagawa Prefectural Central Hospital, Kagawa, Japan; 6 DDS, PhD. Clinical Fellow, Division of Oral and Maxillofacial Surgery, Kagawa Prefectural Central Hospital, Kagawa, Japan; 7 DDS, PhD. Director, Division of Oral and Maxillofacial Surgery, Kagawa Prefectural Central Hospital, Kagawa, Japan

## Abstract

**Background:**

This study aimed to identify (1) the predilection site of postoperative infection after third molar extraction surgery, (2) risk factors associated with postoperative infection, and (3) the cause of the difference between delayed- and early-onset infections.

**Material and Methods:**

This retrospective study included 1010 patients (396 male, 614 female) who had ≥1 third molars extracted (2407; 812 maxilla, 1595 mandible). The risk factors were classified as attributes, general health, anatomic, and operative. Outcome variables were delayed- and early-onset infections.

**Results:**

Postoperative infection was completely absent in the maxilla, and all infections occurred in the mandible, with a probability of 1.94% (31/1595). Bivariate analysis for postoperative infection showed depth of inclusion and intraoperative hemostatic treatment to be significantly associated with the development of infections. Bivariate analysis for delayed- and early-onset infections showed simultaneous extraction of the left and right mandibular third molars to be prominent risk factors.

**Conclusions:**

Postoperative infection occurs mainly in the mandible, and that in the maxilla is very rare. The risk of postoperative infection in the mandible was found to be related to the depth of inclusion and intraoperative hemostatic treatment. Simultaneous extraction of the left and right mandibular third molars appear to increase the risk of delayed-onset postoperative infection.

** Key words:**Third molar extraction surgery, delayed-onset infection, early-onset infection, postoperative infection.

## Introduction

Removal of the third molars is one of the most common surgical procedures in oral and maxillofacial surgery. The reasons for extracting these teeth include acute or chronic pericoronitis, dental crowding, presence of cyst or tumor, periodontal problems, and presence of caries on the adjacent teeth (1) Some complications associated with third molar extractions include postoperative infections (2-6) Although several studies have been published on this subject, patient characteristics and causes of infection remain unclear (5,7,8). To our knowledge, there is no research on the time of infection following third molar extraction surgery, i.e., early- and delayed-onset infections.

The specific aims of this study were to identify 1) the predilection site of postoperative infection following third molar extraction surgery, 2) risk factors associated with postoperative infection following third molar extraction surgery, and 3) the cause of the difference between early- and delayed-onset infections.

## Material and Methods

-Patients

A retrospective study was performed including a total of 1010 patients with 2407 third molars extracted between January and December 2014 at the Division of Oral and Maxillofacial Surgery, Kagawa Prefectural Central Hospital, Kagawa, Japan. Patients who gave their informed consent to extract their third molars, those with ≥1 third molars removed and evidence of postoperative follow-up to assess outcomes, those aged >16 years, and those not presenting any acute-phase symptoms at the time of surgery met the inclusion criteria. Our hospital contacted all the patients who had difficulty following up and instructed them to seek medical attention when any problems occurred. A minimal follow-up period of 6 months was necessary for inclusion in this study. Pregnant women and patients with previous allergic reactions to antibiotics were excluded.

-Surgical procedure

All patients had undergone preoperative panoramic X-ray imaging and CT, if necessary. All procedures were performed under local anesthesia with or without intravenous sedation. The procedures were performed by four expert oral and maxillofacial surgeons at a single institution. The surgical field and all surgical materials were sterilized. The surgeon raised a full-thickness flap, which was protected by a retractor. The British technique of “lingual splits” (9) was not a commonly used surgical technique for mandibular third molar extraction. Sterile low-speed hand pieces and saline solution were used for buccal ostectomy and tooth sectioning when necessary. Abundant irrigation with physiological serum was applied during ostectomy. When hemorrhage occurred during tooth extraction, compression hemostasis with gauze was performed. If hemostasis using gauze was difficult, it was performed using a hemostatic gelatin sponge. The whole third molar tooth was extracted in all cases. Primary wound closure was performed with 4-0 sterile absorbable braided polyglycolic acid sutures (4-0 Surgisorb). Finally, hemostasis was achieved by pressing sterile gauze over the wound, and postoperative measures were administered to each patient.

The same procedure and postoperative clinical management were utilized for all the patients, with a few exceptions. After the operation, patients received antibiotics (cefpodoxime proxetil, 100 mg) every 12 h for 4 days and nonsteroidal anti-inflammatory analgesics celecoxib; 400 mg (for initial pain) or 200 mg every 6 h (for second episode or later) or acetaminophen; 400 mg every 6 h).

-Predictor variables

The predictor variables for the study consisted of sets of exposures considered to be convincingly related to postoperative infection rates, and were classified as attributes, health status, anatomic, and pathological variables. Attributes variables included age and sex. Health status measures included the presence or absence of diabetes, use of corticosteroids, and chronic hepatitis. Anatomical variables included the total number of teeth and the specific tooth type extracted in each patient as well as mandibular third molar position based on the Pell and Gregory (10) and Winter classifications (11). Pathological variables included the presence of radiolucent lesions measuring ≥3 mm that were associated with the lower third molar and presence of adjacent lower second molar caries. Operative variables included intraoperative hemostasis treatment and simultaneous extraction of the left and right mandibular third molars.

-Outcome variables

Delayed-onset infection was defined as an infectious swelling with an onset after 7 days of extraction, whereas early onset was defined as a developing postoperative wound infection within 7 days of surgery.

-Data sampling

All clinical records were examined by the same investigator who also collected the following data: third molar extraction surgery, wound related to postoperative infection, and predictor variables. This study was approved by the Ethics Committee of Kagawa Prefectural Central Hospital (Approval No. 681).

-Statistical analysis

Data were recorded and entered into an electronic database over the course of the study using Microsoft Excel (Microsoft Inc., Redmond, WA, USA). The mean and standard deviations were used where distribution was compatible with normality. Parametric and nonparametric tests (t-test and Pearson chi-square test) were used to compare each group. Any associations on bivariate analyses with *P* <0.05 were included in a multiple logistic regression analysis, which was used to provide adjusted odds ratios (ORs) to control for the simultaneous effects of multiple covariates. The database was transferred to JMP version 11.2 for Macintosh computers (SAS Institute Inc., Cary, NC, USA) for statistical analysis. A *P*-value of <0.05 was considered statistically significant.

## Results

The sample included 1010 patients 396 male (39.2%) and 614 female (60.8%)., with a total of 2407 third molars removed (812 maxilla, 1595 mandible). The mean age of patients was 33.3 ± 14.8 years, and an average number of 2.38 third molar teeth were extracted per patient. All postoperative infections occurred in the mandible 1.94% (31/1595). Early-onset infection occurred in 0.38% (6/1595) of patients and delayed-onset in 1.57% (25/1595). The mean time elapsed from operation to delayed-onset infection was 42.2 days; conversely, that from operation to early-onset infection was only 5.8 days.

A comparison of the distribution of predictor variables according to postoperative infection is summarized in  Table 1. Variables that were statistically associated with postoperative infection (*P* < 0.05) included the depth of inclusion (*P* = 0.008) and intraoperative hemostatic treatment (*P* = 0.0001). The multivariate logistic regression model including these candidate variables found in the bivariate analyses elucidated as being statistically significant (*P* < 0.05). Taken together, the depth of inclusion (OR = B for A, 2.51; *P* = 0.046, C for A, 5.73; P = 0.011), and intraoperative hemostatic treatment (OR = 5.81, *P* = 0.010) was clearly associated with an increased risk for postoperative infection.

**Table 1:**
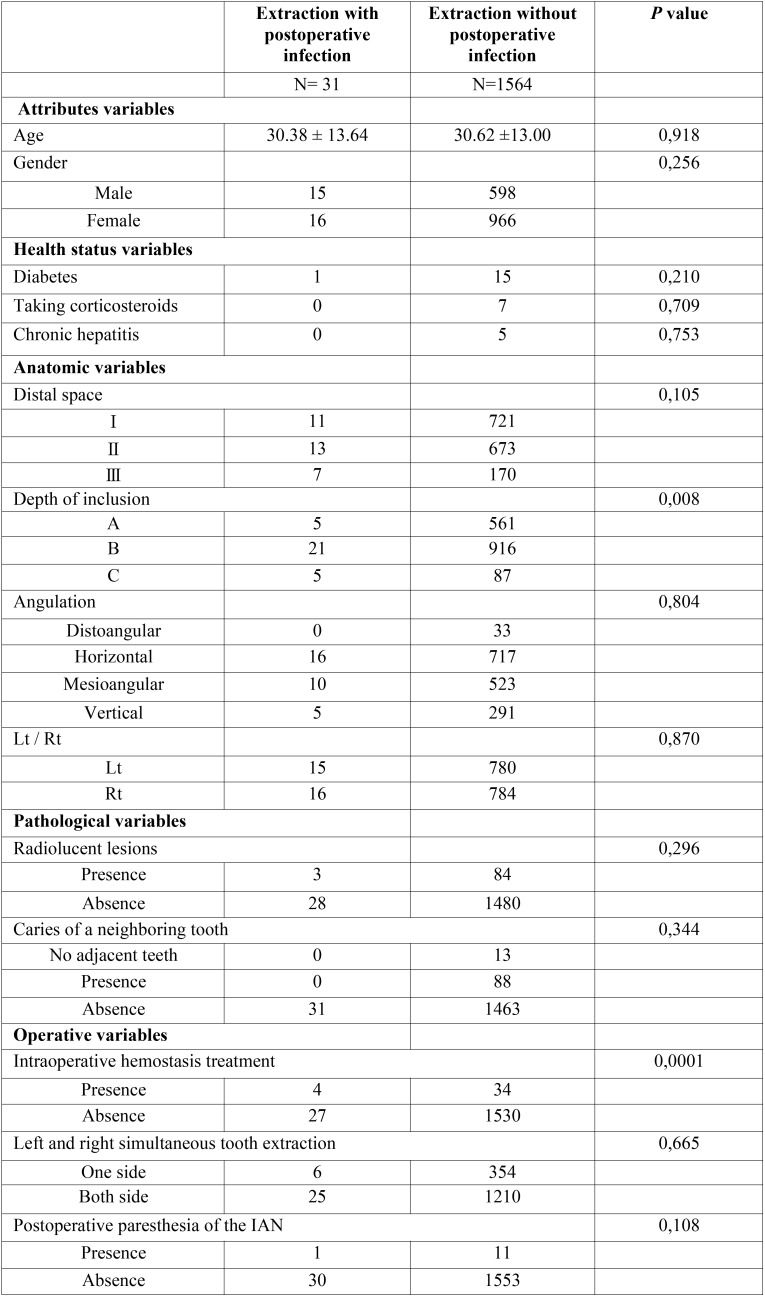
Predictor variables according to postoperative infection.

As a further evaluation, a comparison of the distribution of predictor variables according to delayed- and early-onset infections is summarized in  Table 2. Age (*P* = 0.026) and simultaneous extraction of the left and right mandibular third molars (*P* = 0.001) were statistically associated with complications (*P* < 0.05). The multivariate logistic regression model including these candidate variables found in the bivariate analyses elucidated as being statistically significant (P < 0.05) as well as biologically important variables, including age (OR = 1.06; *P* = 0.127), simultaneous extraction of the left and right mandibular third molars (OR = 20.34; *P* = 0.010). Simultaneous extraction of the left and right mandibular third molars was clearly associated with an increased risk for delayed-onset infection.

**Table 2:**
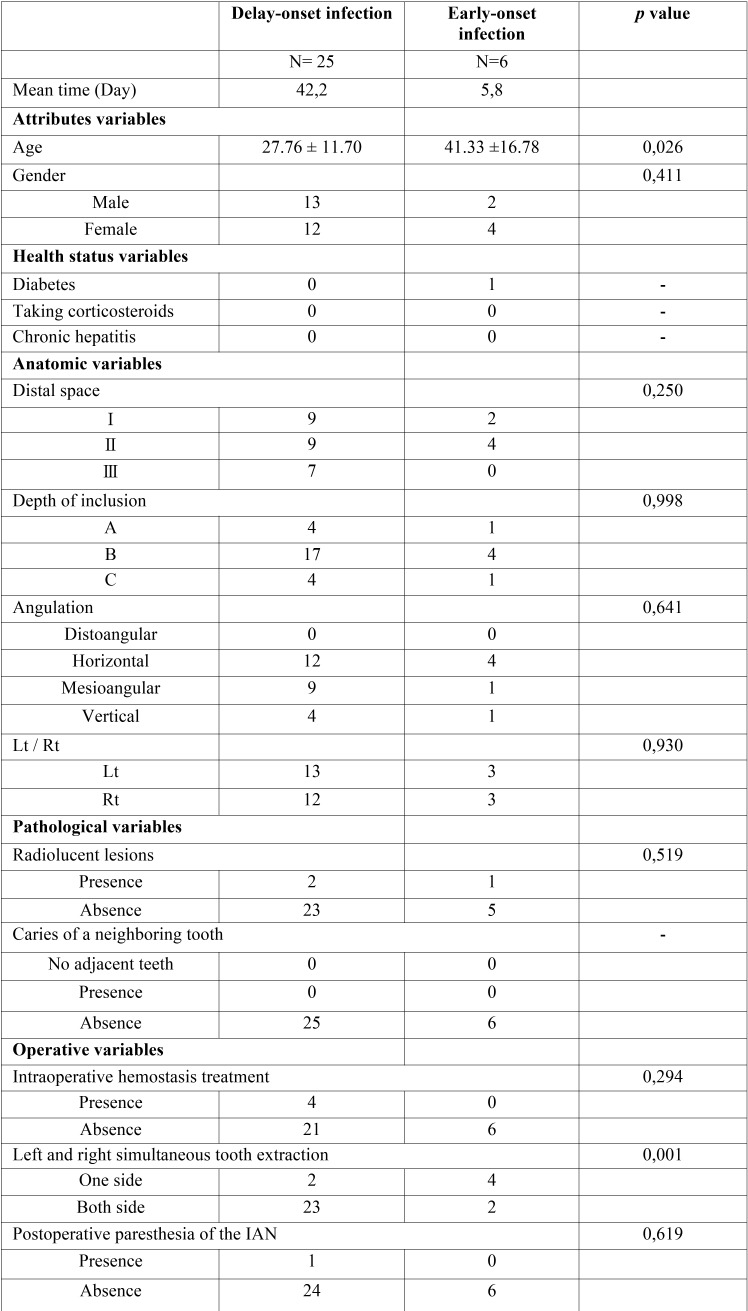
Predictor variables according to delayed- and early-onset infections.

## Discussion

In our study, the overall postoperative infection rate following third molar extraction surgery was 1.28% (31/2407). Importantly, there were no postoperative infections in 812 maxillary third molar extractions; however, the postoperative infection rate was 1.94% (31/1595) for mandibular third molar extractions. The postoperative infection rate recorded in this study was similar to that reported in previous studies which stated that mandibular third molar extractions were more susceptible to infection (1,12) Chiapasco et al. (1) found a postoperative infection rate of 1.5% in the mandible and 0.2% in the maxilla. Clearly, the infection rate was high in the lower jaw, which is similar to our research result. These results implied that mandibular third molar extraction surgery is a risk factor for postoperative infection.

As reported in previous studies, the incidence of postoperative wound infections is approximately 0.4%–6% (3,13-16). However, most complications commonly involve postoperative infection.

Our results as well as those of several past studies found that bone retention was a risk factor for postoperative infection following mandibular third molar extraction surgery (7,17-21). The fact that postoperative infections are more likely to occur in deeply impacted third molars could indicate that surgical invasion, amount of alveolar bone ostectomy, and tooth sectioning are related to the incidence of postoperative infection. In addition, hemostatic treatment during mandibular third molar extraction surgery is also significantly associated with postoperative infections. Generally, postoperative complications following third molar extraction surgery are related to a surgeon’s inexperience and difficult anatomical position of the third molar (2,22). Additionally, postoperative infection of the surrounding bone and soft tissue following mandibular third molar extraction is a common complication that can be reduced with the use of proper surgical techniques. The need for hemostasis during surgery may be due to difficulty in extracting the tooth or operator technique. In addition, a gelatin sponge used for hemostasis may take about 4-6 weeks to absorb (23); therefore, it might promote infection. The association between hemostasis treatment and postoperative infection as a finding of this study is very interesting.

There are a few studies on delayed-onset infections after third molar extractions, and the rates of infections are very similar across studies (0.49%–1.8%), which are also consistent with our results (1.56%; 25/1595) (5,7,17,19) On the other hand, few studies have compared the rates of delayed- and early-onset infections; Christiaens *et al.* (17) reported an early-onset infection rate of 1.0%. Our reported results lower rates in early-onset infection than delayed-onset infection are similar to past study (17).

According to literature, delayed-onset infection more frequently occurs approximately 1 month after surgery (2,8,19,24). In our data, delayed-onset infection occurred at an average of 42.2 postoperative days. Most infections occurred within 3–6 weeks after extraction; therefore, patients should be informed that probability of infection persists even after >1 month of extraction.

Some studies have addressed the incidence and described some clinical features of delayed-onset infection, but there are no clearly identified risk factors (8,17,21). Some reports have considered total mucosal retention, radiotransparent widened follicle, distal space, mandibular third molar angulation, and surgical techniques, such as ostectomy and tooth sectioning, as risk factors for delayed-onset infection (8,17,21); however, these associations were not found in our study. Conversely, our study found that early-onset infection occurred in younger patients more frequently than delayed-onset infection, which is consistent with the results of Osborn *et al.* (3) and Brunello *et al.* (24).

A possible pathway for postoperative infection in mandibular third molar extraction surgery could be the gingival sulcus of the adjacent second molar and hence, Figueiredo *et al.* (8) suggested that total soft tissue retention allows the surgeon to close the wound more tightly; consequently, food impaction can easily introduce bacteria into the wound. In addition, hematomas or food trapped under the flap have been cited as possible causes of delayed-onset infection (19,25). It was considered that the most probable cause of this complication was the dead space created beneath the soft tissue lying behind the distal area of the second molar. According to our study, the mechanism underlying delayed-onset infection was as follows: if it is a closed layer, it cannot completely prevent bacterial invasion. Therefore, food enters the tooth extraction cavity or dead space of the second molar, after which the tooth extraction cavity is completely closed by the very rapid healing of the oral mucous. As a result, infection with anaerobic bacteria is established in approximately 1 month postoperatively. As a further deciding factor, the risk factor for delayed infection was identified in association with simultaneous extraction of the left and right mandibular third molars, and was also a dominant risk factor in logistic regression analysis. The simultaneous extraction of left and right mandibular third molars can cause swelling and trismus, leading to unsanitary conditions in the oral cavity. Particularly in the posterior molar section, the retention of food residue in the extraction tooth cavities strongly supports the abovementioned infection mechanism. This was a very interesting and meaningful clinical discovery in third molar extraction surgery as the most common oral and maxillofacial surgical procedure because this risk factor was not identified in any past studies.

Some bacterial contamination of the area surrounding the tooth extraction is inevitable, from either the patient’s bacterial flora or oral environment. Various therapies are aimed at minimizing the postoperative complications of third molar extraction surgery; of these, the use of systemic antibiotic prophylaxis to avoid postoperative infectious complications following third molar extraction surgery has been widespread but controversial. Prophylactic administration of antibiotics may be an important factor in avoiding infectious complications; however (7,26) there are reports suggesting that systemic prophylactic administration of antibiotics is ineffective (27,28). In our study, we administered systemic prophylactic antibiotics for several days after surgery for all patients; therefore, we could not determine their effect in preventing delayed-onset infections. Therefore, it is necessary to investigate the usefulness of preventive antibiotic administration in the future.

## Conclusions

Infection after the third molar extraction surgery is very rare in the maxilla, but mainly occurs in the mandible. The risk factors of postoperative infection in mandibular third molar extraction were found to be related to the depth of tooth extraction and surgical technique. In addition, delayed-onset infections after third molar extractions are rare. Simultaneous extraction of the left and right mandibular third molars are associated with an increased risk of delayed-onset infection. Therefore, it is necessary for the oral and maxillofacial surgeons to consider these risk factors and the follow-up period following third molar extraction surgery.

## References

[1] Chiapasco M, De Cicco L, Marrone G (1993). Side effects and complications associated with third molar surgery. Oral Surg Oral Med Oral Pathol.

[2] Blondeau F, Daniel NG (2007). Extraction of impacted mandibular third molars: postoperative complications and their risk factors. J Can Dent Assoc.

[3] Osborn TP, Frederickson G, Small IA, Torgerson TS (1985). A prospective study of complications related to mandibular third molar surgery. J Oral Maxillofac Surg.

[4] Reyneke JP, Tsakiris P, Becker P (2002). Age as a factor in the complication rate after removal of unerupted/impacted third molars at the time of mandibular sagittal split ostectomy. J Oral Maxillofac Surg.

[5] Berge TI, Bøe OE (1994). Predictor evaluation of postoperative morbidity after surgical removal of mandibular third molars. Acta Odontol Scand.

[6] Barbosa-Rebellato NL, Thomé AC, Costa-Maciel C, Oliveira J, Scariot R (2011). Factors associated with complications of removal of third molars: a transversal study. Med Oral Patol Oral Cir Bucal.

[7] Piecuch JF, Arzadon J, Lieblich SE (1995). Prophylactic antibiotics for third molar surgery: a supportive opinion. J Oral Maxillofac Surg.

[8] Figueiredo R, Valmaseda-Castellón E, Berini-Aytés L, Gay-Escoda C (2005). Incidence and clinical features of delayed-onset infections after extraction of lower third molars. Oral Surg Oral Med Oral Pathol Oral Radiol Endod.

[9] Brann CR, Brickley MR, Shepherd JP (1999). Factors influencing nerve damage during lower third molar surgery. Br Dent J.

[10] Lima CJ, Silva LC, Melo MR, Santos JA, Santos TS (2012). Evaluation of the agreement by examiners according to classifications of third molars. Med Oral Patol Oral Cir Bucal.

[11] Miclotte A, Franco A, Guerrero ME, Willems G, Jacobs R (2015). The association between orthodontic treatment and third molar position, inferior alveolar nerve involvement, and prediction of wisdom tooth eruption. Surg Radiol Anat.

[12] Bui CH, Seldin EB, Dodson TB (2003). Types, frequencies, and risk factors for complications after third molar extraction. J Oral Maxillofac Surg.

[13] Capuzzi P, Montebugnoli L, Vaccaro MA (1994). Extraction of impacted third molars. A longitudinal prospective study on factors that affect postoperative recovery. Oral Surg Oral Med Oral Pathol.

[14] Herpy AK, Goupil MT (1991). A monitoring and evaluation study of third molar surgery complications at a major medical center. Mil Med.

[15] Momin M, Albright T, Leikin J, Miloro M, Markiewicz MR (2018). Patient morbidity among residents extracting third molars: does experience matter?. Oral Surg Oral Med Oral Pathol Oral Radiol.

[16] Susarla SM, Blaeser BF, Magalnick D (2003). Third molar surgery and associated complications. Oral Maxillofac Surg Clin North Am.

[17] Christiaens I, Reychler H (2002). Complications after third molar extractions: retrospective analysis of 1,213 teeth. Rev Stomatol Chir Maxillofac.

[18] Sisk AL, Hammer WB, Shelton DW, Joy ED (1986). Complications following removal of impacted third molars: the role of the experience of the surgeon. J Oral Maxillofac Surg.

[19] Goldberg MH, Nemarich AN, Marco WP (1985). Complications after mandibular third molar surgery: a statistical analysis of 500 consecutive procedures in private practice. J Am Dent Assoc.

[20] Benediktsdóttir IS, Wenzel A, Petersen JK, Hintze H (2004). Mandibular third molar removal: risk indicators for extended operation time, postoperative pain, and complications. Oral Surg Oral Med Oral Pathol Oral Radiol Endod.

[21] Figueiredo R, Valmaseda-Castellón E, Berini-Aytés L, Gay-Escoda C (2007). Delayed-onset infections after lower third molar extraction: a case-control study. J Oral Maxillofac Surg.

[22] Nguyen E, Grubor D, Chandu A (2014). Risk factors for permanent injury of inferior alveolar and lingual nerves during third molar surgery. J Oral Maxillofac Surg.

[23] Duenas-Garcia OF, Goldberg JM (2008). Topical hemostatic agents in gynecologic surgery. Obstet Gynecol Surv.

[24] Brunello G, De Biagi M, Crepaldi G, Rodrigues FI, Sivolella S (2017). An observational cohort study on delayed-onset infections after mandibular third-molar extractions. Int J Dent.

[25] Goldberg MH, Galbraith DA (1984). Late onset of mandibular and lingual dysesthesia secondary to postextraction infection. Oral Surg Oral Med Oral Pathol.

[26] Lacasa JM, Jiménez JA, Ferrás V, Bossom M, Sóla-Morales O, García-Rey C (2007). Prophylaxis versus pre-emptive treatment for infective and inflammatory complications of surgical third molar removal: a randomized, double-blind, placebo-controlled, clinical trial with sustained release amoxicillin/clavulanic acid (1000/62.5 mg). Int J Oral Maxillofac Surg.

[27] Poeschl PW, Eckel D, Poeschl E (2004). Postoperative prophylactic antibiotic treatment in third molar surgery--A necessity?. J Oral Maxillofac Surg.

[28] Siddiqi A, Morkel JA, Zafar S (2010). Antibiotic prophylaxis in third molar surgery: a randomized double-blind placebo-controlled clinical trial using split-mouth technique. Int J Oral Maxillofac Surg.

